# Exosome and Melatonin Additively Attenuates Inflammation by Transferring miR-34a, miR-124, and miR-135b

**DOI:** 10.1155/2020/1621394

**Published:** 2020-11-24

**Authors:** June Seok Heo, Ja-Yun Lim, Dae Wui Yoon, Sangshin Pyo, Jinkwan Kim

**Affiliations:** ^1^Cell Therapy Center, Severance Hospital, Seoul 03722, Republic of Korea; ^2^Department of Integrated Biomedical and Life Sciences, Graduate School, Korea University, Seoul 02841, Republic of Korea; ^3^Department of Biomedical Laboratory Science, College of Health Science, Jungwon University, Goesan 28024, Republic of Korea; ^4^Department of Clinical Laboratory Science, Daejeon Institute of Science and Technology, Daejeon 35408, Republic of Korea

## Abstract

The positive effects of mesenchymal stem cells (MSCs) are primarily activated through molecular secretions known as paracrine activity, which regulates the function of various cell types including immune cells. Accumulating evidence shows that exosomes of soluble factors released from MSCs are potential alternative agents for stem cell-based therapy, although the exact underlying mechanism has not been elucidated. The purpose of this study was to evaluate the potential effects of exosomes produced by adipose-derived MSCs and to examine the changes in anti-inflammatory genes in concurrence with the polarization of M2 macrophages in cellular models ex vivo. Isolated exosomes were used to investigate the inflammatory modulation in pro-inflammatory cytokine-treated fibroblasts and THP-1 cells. The anti-inflammatory mRNA expression associated with M2 macrophages was significantly upregulated after exosome treatment in an interferon gamma and tumor necrosis factor alpha-treated inflammatory environment. Furthermore, melatonin-stimulated exosomes exerted superior anti-inflammatory modulation via exosomal miRNAs miR-34a, miR-124, and miR-135b, compared with exosomes. Our results indicate that melatonin-stimulated exosomes originating from adipose-derived MSCs are safe and efficient tools for regenerative medicine to treat inflammatory diseases.

## 1. Introduction

Mesenchymal stem cells (MSCs) are promising cell sources owing to their multipotent and immunosuppressive properties [1]. Accumulating evidence shows that MSCs exert anti-inflammatory effects by modulating immune cells and the inflammatory environment [2]. Hence, clinical applications using various types of MSCs have been actively conducted to develop new treatments, especially for inflammation-related disorders such as graft-versus-host disease, multiple sclerosis, joint diseases, inflammatory bowel diseases, inflammatory airway disease, and pulmonary diseases [3]. Although the effectiveness of MSCs is predominantly associated with paracrine effects rather than direct differentiation or engraftment, the underlying mechanisms of MSC therapy are unclear [4].

Recently, the use of bioactive molecules released from MSCs has been described as a novel treatment strategy because of its low tumorigenicity and immunogenicity [5]. Among the trophic factors of MSCs, exosomes have attracted much attention as new mediators in cell-to-cell interactions [6]. Exosomes, one of the extracellular vesicles secreted by cells, are cup-shaped lipid-bilayer nano-sized vesicles (40–120 nm). Exosomes originating from the endosomal pathway contain biological factors such as proteins, lipids, and nucleic acids that mediate intercellular communication [7]. MSC-derived exosomes can promote phenotypic and functional changes in the surrounding cells and microenvironment by inducing the activation of regenerative events [8]. Recent studies have also demonstrated that exosomes play important roles in the regulation of inflammatory phenotypes via M2 macrophage polarization [9, 10]. Therefore, an exact understanding of the immunomodulatory effects of exosomes is essential to develop more efficient therapeutic strategies for curing intractable or chronic inflammatory diseases.

It has been demonstrated that MSC-derived exosomes can upregulate anti-inflammatory factors IL-10 and TGF-*β* and downregulate inflammatory factors IL-6 and TNF*α* [11, 12]. In the present study, we examined the anti-inflammatory properties of exosomes secreted from adipose MSCs, using fibroblasts and THP-1 cells treated with pro-inflammatory cytokines interferon gamma (IFN*γ*) and tumor necrosis factor alpha (TNF*α*). We conducted experiments to investigate whether the exosomes could induce anti-inflammatory environments in these models.

## 2. Materials and Methods

### 2.1. Flow Cytometry

Human adipose tissue-derived MSCs were obtained from healthy donors with the approval from the research ethics committee of Severance Hospital of Yonsei University, Seoul, Republic of Korea, following informed consent (approval no. 4-2019-0060). For surface marker analysis, cultured MSCs were harvested as single cells and incubated with fluorescein isothiocyanate (FITC) or phycoerythrin- (PE-) conjugated antibodies (BD Biosciences Pharmingen, San Diego, CA, USA). The antibodies used in this study were anti-CD14, anti-CD29, anti-CD31, anti-CD34, anti-CD44, anti-CD45, anti-CD73, anti-CD90, anti-CD105, and anti-CD106. Data were acquired using a flow cytometer (Cytomics Flow Cytometer, Beckman Coulter, Fullerton, CA, USA). FITC- and PE-conjugated isotype control antibodies were used as negative controls.

### 2.2. Isolation of Exosomes

Exosomes were isolated from MSCs using the ExoQuick-TC exosome precipitation reagent, according to the manufacturer's protocols (System Biosciences, CA, USA). Briefly, 5 mL culture medium was transferred to a 15 mL conical tube after centrifugation at 1500 × *g* for 5 min at RT to remove dead cells and cell debris. Thereafter, the 1 mL ExoQuick-TC reagent was added to the medium supernatant and mixed via four inversions. The mixture was incubated overnight at 4°C. The next day, the mixture was centrifuged at 1500 × *g* for 30 min to pellet the exosomes. The exosome pellet was suspended in phosphate-buffered saline after removing the supernatant. Following quantification using a BCA protein assay kit (Invitrogen, Carlsbad, CA, USA), exosomes were stored at −80°C until use. Ten micrograms per milliliter of exosomes was used for the experiments. The morphology of isolated exosomes was observed using transmission electron microscopy (TEM) (JEM-1011, JEOL, Japan). Briefly, a formvar-carbon-coated EM grid was placed formvar-side down on top of an exosome drop for 1 min. Thereafter, the grid was placed on a drop of 2% uranyl acetate and blotted with filter paper. Finally, excess uranyl acetate was removed.

### 2.3. Western Blotting

The western blot assay was performed as reported previously [9]. Briefly, following boiling at 95°C for 5 min, exosomes were separated on 12% gradient precast gels via sodium dodecyl sulfate polyacrylamide gel electrophoresis and transferred onto a polyvinylidene difluoride membrane (Bio-Rad Laboratories, Redmond, WA, USA). Following blocking with 5% skim milk in Tris-buffered saline with Tween, the membrane was incubated overnight at 4°C with the primary antibodies CD9 (Abcam, Cambridge, MA, USA; 1 : 400) and CD63 (Abcam; 1 : 500). The next day, the membrane was incubated with HRP-conjugated secondary antibodies and anti-mouse and anti-rabbit IgG (Invitrogen; 1 : 1000) at RT for 1 h. Following incubation with enhanced chemiluminescence substrate (Bio-Rad) for 1 min, the expression was detected using an LAS4000 mini system (GE Healthcare, Uppsala, Sweden).

### 2.4. Cell Proliferation Assay

Fibroblasts were seeded at a density of 1 × 10^3^/well in a 96-well plate (BD Falcon, USA) to analyze cell proliferation. When cells were plated, IFN*γ* and TNF*α* were added to each well (10–40 ng/mL). As previously described, the growth activity was determined using a WST-8-based EZ-Cytox kit (Daeil Lab, Seoul, Korea) [13]. Two days later, cell proliferation was analyzed according to the manufacturer's instructions. Briefly, the EZ-Cytox reagent was added to each well and incubated for 3 h. Following shaking for 1 min, the absorbance was measured at 450 nm using a microplate reader (Molecular Devices, San Jose, CA, USA). Untreated cells were used as controls. To optimize the concentration of melatonin, MSCs were treated with varying concentrations (0.5–50 *μ*M) of melatonin. Proliferation rates were evaluated using an EZ-Cytox kit after 3 d. Untreated cells (0 *μ*M of melatonin) were used as controls.

### 2.5. Real-Time PCR Assay

Quantitative PCR was performed as described previously [9]. Briefly, total RNA was isolated using the RiboEx reagent (GeneAll, Seoul, Korea). Extracted RNA was reverse transcribed into cDNA using Maxime RT PreMix, according to the manufacturer's protocols (iNtRON, Seongnam, Korea). PCR was performed using the LightCycler 480 SYBR Green I Master mix on a LightCycler 480 System under the following conditions: 95°C for 5 min, 95°C for 10 s, 45 cycles of 60°C for 20 s, and 72°C for 15 s. The primer sequences used are listed in Table 1. Differences between the crossing point (Cp) values of glyceraldehyde 3-phosphate dehydrogenase (GAPDH) and target genes were used to calculate *Δ*Cp values. Relative expression levels were determined using the following formula: relative expression level = 2^−(SΔCp − CΔCp)^. For miRNA reactions, PCR was performed at 95°C for 10 min, followed by 40 cycles of 95°C for 15 s, 60°C for 1 min, and 72°C for 10 s. U6 snRNA was used as an internal control.

### 2.6. Statistical Analysis

Data are expressed as the mean ± standard deviation. Statistical analysis was performed using SPSS 18 (SPSS Inc., Chicago, IL, USA). Data were analyzed using one-way analysis of variance followed by Bonferroni's correction or Student's *t*-test. Statistical significance was set at *P* < 0.05.

## 3. Results

### 3.1. Identification of MSCs and Exosomes

**Figure 1:**
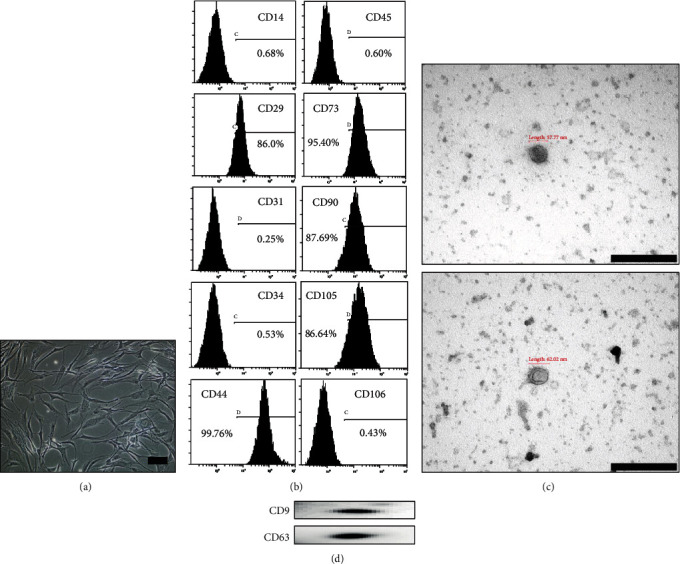
Isolation and characterization of adipose MSCs and adipose MSC-derived exosomes. (a) Spindle-shaped fibroblast-like morphology observed via an inverted microscope (×100 magnification). (b) Surface markers of cultured cells showing the typical pattern of MSCs. (c) TEM image of isolated exosomes (scale bar = 200 nm). (d) The expression of CD9 and CD63 in exosomes was detected using western blotting. A representative independent experiment is shown. MSCs: mesenchymal stem cells; TEM: transmission electron microscopy.

MSCs were isolated from the adipose tissue of healthy human donors. Cultured MSCs were examined and photographed under an inverted microscope. The adherent cells exhibited spindle fibroblast-like morphology (Figure 1(a)). Flow cytometry results showed that the cells were positive for MSC markers CD29, CD44, CD73, CD90, and CD105, but not for hematopoietic/endothelial markers CD14, CD31, CD34, CD45, and CD106 (Figure 1(b)). These results demonstrated that the cultured cells showed typical MSC characteristics.

Subsequently, exosomes isolated from MSCs were characterized using TEM and western blotting. Morphological characterization showed the existence of small membrane vesicles measuring 57.77–62.02 nm (Figure 1(c)). Western blotting revealed that the isolated vesicles were positive for the expression of exosomal markers such as CD9 and CD63 (Figure 1(d)). TEM and western blotting results revealed that the vesicles isolated from MSCs were exosomes.

### 3.2. Effects of Exosomes on IFN*γ* and TNF*α*-Treated Fibroblasts

**Figure 2:**
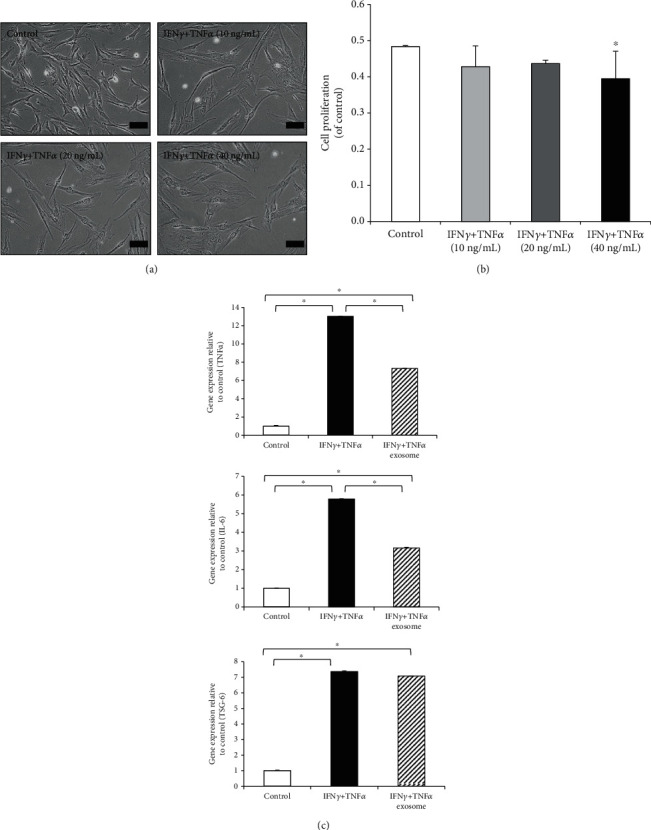
Treatment with combination of IFN*γ* and TNF*α* to induce inflammation in fibroblasts. (a) Optical images of MSCs stimulated with IFN*γ* and TNF*α* at concentrations of 10, 20, and 40 ng/mL for 48 h. A representative image based on three independent experiments is shown (×200 magnification). (b) Cell proliferation evaluated using the WST-1-based colorimetric assay. ^∗^*P* < 0.05 compared with the control group. (c) mRNA expression levels of inflammation-associated genes *TNFα*, *IL6*, and *TSG6*, analyzed using real-time PCR. Data are represented as the mean ± SD of three independent experiments. IFN*γ*: interferon gamma; TNF*α*: tumor necrosis factor alpha; MSCs: mesenchymal stem cells.

To determine whether pro-inflammatory cytokines affect the morphology and proliferation activity of fibroblasts, cells were cultured with IFN*γ* and TNF*α* at concentrations from 10 to 40 ng/mL for 48 h. We observed that treatment with pro-inflammatory cytokines induced an enlarged senescent-like morphology at high concentrations (40 ng/mL IFN*γ* and 40 ng/mL TNF*α*) (Figure 2(a)). Moreover, the concentration of 40 ng/mL cytokines resulted in the lowest growth rate of fibroblasts (Figure 2(b)). Therefore, this concentration was selected to induce inflammation.

To evaluate the effects of exosomes on pro-inflammatory cytokine-treated fibroblasts, the inflammation-related gene expression was analyzed using real-time PCR. Our data show that the expression levels of pro-inflammatory cytokines TNF*α* and IL-6 genes increased in the presence of cytokines and decreased upon exosome (10 *μ*g/mL) treatment (Figure 2(c)). Alternatively, the gene expression level of the anti-inflammatory factor TSG-6 did not change despite exosome treatment (Figure 2(c)). These results indicate that exosomes exert anti-inflammatory effects by downregulating pro-inflammatory genes.

### 3.3. Effects of Exosomes on Proinflammatory Cytokine-Treated THP-1 Cells

**Figure 3:**
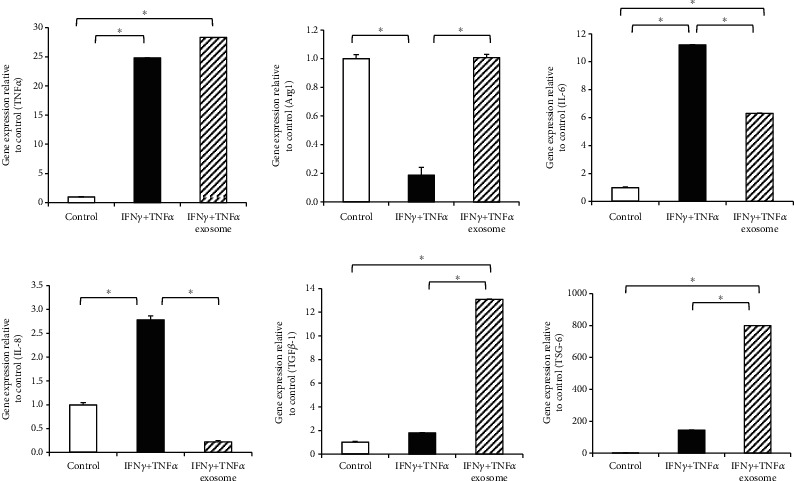
Effects of exosomes on pro-inflammatory cytokine-treated THP-1 cells. mRNA expression levels after pro-inflammatory cytokine (IFN*γ* and TNF*α*) treatment were determined using real-time PCR and normalized with GAPDH mRNA levels. Untreated THP-1 cells were used as the control. Data are represented as the mean ± SD of three independent experiments ^∗^*P* < 0.05. IFN*γ*: interferon gamma; TNF*α*: tumor necrosis factor alpha; GAPDH: glyceraldehyde 3-phosphate dehydrogenase.

THP-1 cells are a human monocytic cell line derived from an acute monocytic leukemia patient. THP-1 cells respond to diverse inflammatory cytokines and are used as an inflammation model in vitro [14]. To assess the effects of exosomes on inflammation in greater detail, we used the THP-1 cell line. Stimulation with IFN*γ* and TNF*α* increased the expression of TNF*α*, IL-6, and IL-8, which are associated with pro-inflammatory M1 macrophages; however, the expression of TGF- *β*1 and TSG-6, which are related to anti-inflammatory M2 macrophages, was significantly enhanced only in the presence of exosomes (Figure 3). Collectively, these results indicate that exosomes can inhibit inflammatory responses induced by IFN*γ* and TNF*α*.

### 3.4. Effects of Melatonin on MSC Proliferation and Characterization of Melatonin-Stimulated Exosomes (Mel-Exosome)

**Figure 4:**
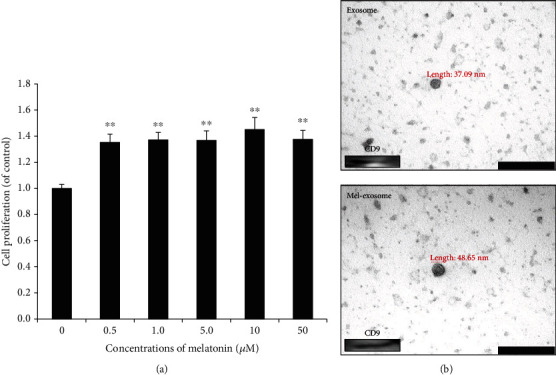
Effects of melatonin on the MSC growth activity and exosome characterization. (a) MSCs were cultured with different concentrations (0-50 *μ*M) of melatonin for 72 h. The proliferation rates of cells were evaluated using the proliferation assay kit (EZ-cytox assay). Data are represented as the mean ± SD of three independent experiments ^∗∗^*P* < 0.01. (b) The morphology of untreated exosomes and melatonin-stimulated exosomes (Mel-Exosome) was confirmed by TEM (scale bar = 200 nm). The exosomal marker CD9 was detected in untreated exosomes and melatonin-stimulated exosomes (Mel-Exosome) by western blotting. A representative independent experiment is shown. MSC: mesenchymal stem cell; TEM: transmission electron microscopy.

Different concentrations (0–50 *μ*M) of melatonin were applied to cells to investigate its effects on THE MSC proliferation activity and its optimal concentration. Overall, the growth rates of cells increased after melatonin treatment regardless of concentration (Figure 4(a)). At varying concentrations, 10 *μ*M of melatonin resulted in the highest growth rate after 3 d of culture (Figure 4(a)). These results are in line with those of a previous study [15]. Therefore, melatonin at 10 *μ*M was used as the optimal concentration.

To identify changes depending on melatonin treatment, the exosome morphology derived from Mel-Exosome and untreated cells was confirmed by TEM (Figure 4(b)). The size of isolated exosomes was in the range of 30–50 nm, and the CD9 exosomal marker was detected in both Mel-Exosome and untreated exosomes, indicating that exosomes were not influenced by melatonin treatment (Figure 4(b)).

### 3.5. Effects of Melatonin-Stimulated Exosomes on Pronflammatory Cytokine-Treated THP-1 Cells

**Figure 5:**
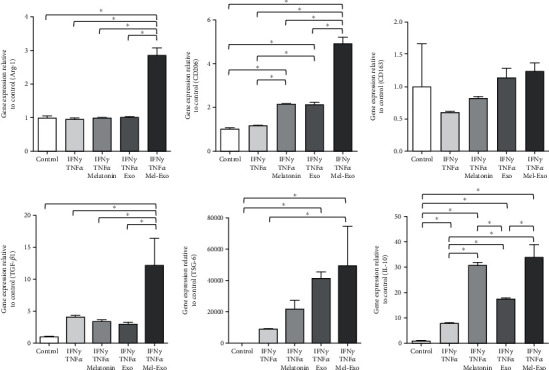
Effects of untreated exosomes and melatonin-stimulated exosomes (Mel-Exosome) on pro-inflammatory cytokines-treated THP-1 cells. Relative mRNA expression levels after pro-inflammatory cytokine (IFN*γ* and TNF*α*) treatment were determined using real-time PCR and normalized with GAPDH mRNA levels. Untreated THP-1 cells were used as control. Data are represented as the mean ± SD of three independent experiments. ^∗^*P* < 0.05. IFN*γ*: interferon gamma; TNF*α*: tumor necrosis factor alpha; GAPDH: glyceraldehyde 3-phosphate dehydrogenase.

Following treatment with 40 ng/mL IFN*γ* and TNF*α*, THP-1 cells were equivalently treated with 10 *μ*M, 10 *μ*g/mL exosomes, and 10 *μ*g/mL melatonin (10 *μ*M)-Mel-Exosome to evaluate the ability of melatonin-stimulated exosomes. First, we evaluated macrophage differentiation from THP-1 cells by real-time PCR. We detected elevated gene expression levels of M2 macrophage markers CD206 and CD163 under all conditions (Figure 5). Notably, we observed that their expression levels were significantly upregulated in melatonin-treated exosomes (Figure 5). However, only melatonin-treated exosomes (Mel-Exosome) were able to upregulate the expression of the M2 macrophage marker Arg-1 (Figure 5).

Second, we detected the expression levels of anti-inflammatory genes TGF-*β*1, TSG-6, and IL-10. We noticed that the TSG-6 gene expression in the exosomes and Mel-Exosome groups significantly increased, and that of TGF-*β*1 was only markedly expressed in melatonin-treated exosomes (Figure 5). Notably, the IL-10 gene expression was significant only after melatonin and Mel-Exosome treatment, indicating that the IL-10 expression was affected by melatonin (Figure 5). Collectively, our data showed that exosomes could induce M2 polarization in agreement with anti-inflammatory changes, and Mel-Exosome could strongly promote these effects, implying their applicability in inflammation-related diseases.

**Figure 6:**
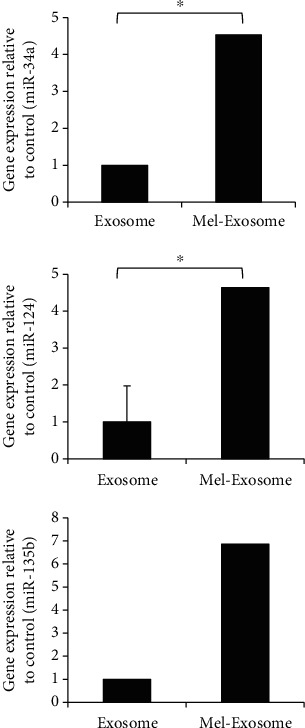
Expression of miR-34a, miR-124, and miR-135b in exosomes. The relative expression levels of miRNAs were analyzed in melatonin-stimulated exosomes (Mel-Exosome) compared with untreated exosomes by real-time PCR. Data are represented as the mean ± SD of three independent experiments. ^∗^*P* < 0.05.

Finally, we analyzed specific miRNAs miR-34a, miR-124, and miR-135b of anti-inflammatory M2 macrophages between exosomes and Mel-Exosome. As shown in Figure 6, the expression of miR-34a, miR-124, and miR-135b significantly increased in melatonin-stimulated exosomes compared with untreated exosomes. The results of these studies verify that melatonin-stimulated exosomes (Mel-Exosome) have powerful effects on M2 polarization-mediated anti-inflammation.

## 4. Discussion

The therapeutic effects of MSCs are associated with the transfer of their paracrine factors, which regulate cellular functionality [16]. Previously, Jeong et al. showed that transplantation of cultured MSCs can form tumors with chromosomal abnormalities in vivo [17]. An important advantage of therapy using exosomes is freedom from clinical risks of transplanted cells due to lower immunogenicity and no risk of tumorigenicity [5]. Recent evidence has shown that adipose-derived MSC exosomes induce M2 macrophages with an anti-inflammatory phenotype [9]. Furthermore, several studies also indicate that MSC-derived exosomes exert anti-inflammatory effects by enhancing anti-inflammatory factors such as IL-10 and TGF-*β*1 [18, 19]. Based on previous reports, we investigated the potential effects of exosomes derived from adipose MSCs in IFN*γ* and TNF-*α*-treated inflammatory models. Notably, exosomes markedly attenuated inflammation-related genes, including TNF*α* and IL-6, in pro-inflammatory cytokine-induced fibroblasts. In a pro-inflammatory cytokine-stimulated THP-1 cellular model, exosome treatment resulted in the notable induction of an M2 macrophage-mediated anti-inflammatory environment. The changes in the expression of inflammation-related markers coincided with an increase in the expression of Arg-1 and TSG-6 of anti-inflammatory M2 macrophages, suggesting that exosomes could be used as therapeutic agents to control inflammation in the biomedical field.

Melatonin has been reported to play crucial roles in stem cell research [20]. For example, melatonin has been demonstrated to promote stem cell proliferation and therapeutic efficacy by regulating inflammatory conditions [21–23]. However, the synergistic effect of combined melatonin and exosome has been not extensively studied. MSCs stimulated by pro-inflammatory cytokines secrete soluble molecules known as paracrine factors, which promote M2 macrophage polarization expressing Arg-1, CD206, and CD163. Polarized M2 macrophages facilitate anti-inflammatory responses by enhancing TGF-*β*1, TSG-6, and IL-10 [24]. To investigate the effects of melatonin-stimulated exosomes on inflammation, we evaluated the expression of specific genes associated with anti-inflammatory M2 macrophages. The relative gene expression demonstrated that in comparison with untreated exosomes, M2 macrophage-mediated anti-inflammatory changes were reinforced after melatonin-stimulated exosome application. Alternatively, some results showed that exosomes failed to induce substantial anti-inflammatory M2 macrophages. These results might be attributable to incomplete macrophage polarization, consistent with the conclusions of previous findings [9]. It has been reported that melatonin enhances the IL-10 expression to suppress inflammation [25]. Our data demonstrated that the IL-10 expression significantly increased only by melatonin treatment. Therefore, we believe that melatonin-stimulated exosomes have a synergistic effect on the inflammatory environment.

An increasing number of reports indicate that exosome-delivered miRNAs regulate inflammatory responses by targeting mRNAs [26]. To determine whether exosomes exert M2 macrophage-mediated anti-inflammatory effects via specific miRNAs, we analyzed the expression of miRNAs in melatonin-stimulated miRNAs and compared them with those in untreated exosomes. MiR-34a has been reported to promote M2 macrophages and miR-124 reduced M1 polarization and enhanced M2 polarization [27, 28]. In addition, the anti-inflammatory properties of MSCs have been linked to miR-135b-mediated M2 polarization [29]. Thus, we confirmed that miR-34a, miR-124, and miR-135b were significantly upregulated in melatonin-stimulated exosomes. The upregulation of these miRNAs strongly supports a significant increase in Arg-1, CD206, CD163, TGF-*β*1, TSG-6, and IL-10 gene expression, which contributed to the promotion of an anti-inflammatory microenvironment. Hence, our data suggest that melatonin-stimulated exosomes may be a promising therapeutic tool to suppress inflammation caused by chronic inflammatory diseases or autoimmune disorders, even if the mechanisms are not fully understood.

## 5. Conclusions

Inflammation is a basic pathological mechanism of complex biological responses in a variety of diseases. The inflammatory reaction involves complex interactions of macrophages, neutrophils, and lymphocytes, which are called inflammatory cells [30]. Although exosomes play a crucial role in modulating inflammatory responses, the mechanisms and signaling pathways related to the activation or inactivation of diverse inflammatory mediators and cytokines remain unclear. Moreover, additional confirmatory data need to be acquired using miRNA sequencing and analysis at the protein level. Additionally, further investigations are necessary to explore the synergistic effect of combined melatonin and exosomes using animal models. In conclusion, the findings of the present study contribute to the development of stem cell-based cell-free therapies by providing valuable information that can be used for inflammation-related diseases.

## Figures and Tables

**Table 1:** Primers used for real-time PCR.

Name	Sequence
GAPDH	F: ACCCACTCCTCCACCTTTGA R: CTGTTGCTGTAGCCAAATTCGT
IL-6	F: AGACAGCCACTCACCTCTTCAG R: TTCTGCCAGTGCCTCTTTGCTG
IL-8	F: AAGAGAGCTCTGTCTGGACC R: GATATTCTCTTGGCCCTTGG
TSG-6	F: GGTGTGTACCACAGAGAAGCA R: GGGTTGTAGCAATAGGCATCC
TGF-ß1	F: TACCTGAACCCGTGTTGCTCTC R: GTTGCTGAGGTATCGCCAGGAA
CD163	F: CGGCTGCCTCCACCTCTAAGT R: ATGAAGATGCTGGCGTGACA
TNF*α*	F: TTGAGGGTTTGCTACAACATGGG R: GCTGCACTTTGGAGTGATCG
Arg-1	F: ACAGTTTGGCAATTGGAAGCA R: CACCCAGATGACTCCAAGATCAG
CD206	F: TTCGGACACCCATCGGAATTT R: CACAAGCGCTGCGTGGAT
IL-10	F: TCTCCGAGATGCCTTCAGCAGA R: TCAGACAAGGCTTGGCAACCCA
U6	F: CTCGCTTCGGCAGCACATATACT R: ACGCTTCACGAATTTGCGTGTC
miR-34a	F: ACACTCCAGCTGTGACTGGTTGACCAGA R: CTCAACTGGTGTCGTGGA
miR-124	F: TAAGGCACGCGGTGAATGCC R: GATTGAATCGAGCACCAGTTAC
miR-135b	F: GCTTATGGCTTTTCATTCCT R: GTGCAGGGTCCGAGGT

## Data Availability

The data used to support the findings of this study are available from the corresponding author upon request.
